# The influence of family social support on quality of life of informal caregivers of cancer patients

**DOI:** 10.1002/nop2.887

**Published:** 2021-05-05

**Authors:** Margarita García‐Carmona, Francisco García‐Torres, Marcin Jacek Jabłoński, Ángel Gómez Solís, María José Jaén‐Moreno, Juan A. Moriana, María José Moreno‐Díaz, Enrique Aranda

**Affiliations:** ^1^ Department of Psychology University of Cordoba Cordoba Spain; ^2^ IMIBIC Health Research Institute/Reina Sofía University Hospital of Cordoba Cordoba Spain; ^3^ Institute of Psychology Faculty of Philosophy Jesuit University Ignatianum in Krakow Krakow Poland; ^4^ Reina Sofía University Hospital of Cordoba Cordoba Spain; ^5^ IMIBIC Health Research Institute/Department of Social Health Sciences Radiology and Physical Medicine University of Córdoba Córdoba Spain; ^6^ Department of Social Health Sciences, Radiology and Physical Medicine University of Córdoba Córdoba Spain; ^7^ Medical Oncology Department Reina Sofía University Hospital Córdoba Spain

**Keywords:** anxiety, cancer, caregiver, depression, quality of life, social support

## Abstract

**Aim:**

Caregivers of cancer patients are at high risk of experiencing impairments in terms of anxiety, depression and quality of life. This study examines the mediation capacity that perceived emotional support can have after diagnosis and six months later between depression and anxiety after diagnosis and quality of life in informal caregivers of cancer patients.

**Design:**

A sample of 67 informal caregivers of cancer patients was used. This study is longitudinal, ex post facto prospective, with convenience sampling.

**Methods:**

Participants completed the Medical Outcomes Study 36‐Item Short Form (SF‐36), the Hospital Anxiety and Depression Scale (HADS) and the Berlin Social Support Scale (BSSS) and a sociodemographic questionnaire. Data were collected between March 2017 and November 2018.

**Results:**

Spearman's correlation analysis showed that anxiety, depression and perceived emotional support were related to quality of life. The mediation analysis showed that the relationship between depression after diagnosis and quality of life six months later was mediated by perceived emotional support.

## 1 INTRODUCTION

Cancer is still one of the principal causes of morbidity and mortality around the world, second only to cardiovascular disease. It is estimated that in 2018, the deaths caused by cancer reached 9.6 million, with 18.1 million new cancer diagnoses, showing an increasing trend, as in 2012 the cancer incidence was 14 million worldwide (Bray et al., 2018). In Spain, the data show a similar pattern, with cancer as the cause of 427,721 deaths, and the new cancer diagnosis rate keeps growing from 247,721 in 2015–277,234 in 2019 (Sociedad española de Oncología Médica [SEOM], 2020).

Patients diagnosed with cancer experience an alteration in their life course, modifying some levels of their lives: physical, psychological, social, work and economic. This situation also affects their families or close relatives who act as caregivers (Asociación Española Contra el Cáncer [AECC], 2019; Stanton et al., 2015). An informal caregiver can be defined as an individual who provides care to the cancer patient and is not paid to do so, usually without previous training in different areas, including physical, emotional, and financial. This situation may lead to impairments in terms of psychological distress and lower quality of life, particularly in the first months after cancer diagnosis (American Cancer Society [ACS], 2016; McDonald et al., 2018; Moreno‐González et al., 2019; Sklenarova et al., 2015; Tan et al., 2018). Caregivers often have higher levels of distress than the patients, comprising 69.1% versus 54.1% the population with distress, respectively, using The National Comprehensive Cancer Network Distress Thermometer (DT) and a cut‐off point of 5 points (Sklenarova et al., 2015). Physical health in caregivers is maintained during the care period, but depressive and anxiety disorders are the most prevalent psychiatric diagnosis at baseline, although at three months and six months of follow‐up they improved (Lee et al., 2018). Quality of life is also affected in this population, as previous research showed that 58% of caregivers have worse quality‐of‐life scores compared to the normal population (Bauer et al., 2018). Quality of life can be described as a multidimensional term that includes health and well‐being as central elements (Sánchez et al., 2015), focusing on different aspects such as physical function, physical role, body pain, general health, vitality, social function, emotional role and mental health (Vilagut et al., 2005). Despite the relevance of studying quality of life in this context, few studies have focused on the quality of life in caregivers in the first months after their family member cancer diagnosis (Peh et al., 2020; Lambert et al., 2017). Previous research reports that caregivers of cancer patients with haematopoietic stem cell transplantation show significant decline in quality‐of‐life scores of vitality, social functioning and emotional role during hospitalization (El‐Jawahri et al., 2015). Anxiety and depression are identified as factors that influence quality of life; that is, a high level of these symptoms is related to worse quality of life. On the other hand, caregivers who present a high level of distress and a poorer quality of life in the long term, especially in the mental health dimension, also presented increased demands in patient care and/or unmet psychosocial needs (Kim et al., 2016; Li et al., 2016; Qiuping et al., 2018). Therefore, caregivers who experience intense emotional and physiological stress during the care process are more vulnerable to physical and psychological health problems (De Padova et al., 2019).

Besides anxiety and depression, social support appears as a factor that influences quality of life in caregivers. It is well established that the lack of social support is negatively related to a poorer quality of life over time, suggesting that social support affects not only the mental health of caregivers but also their physical health (Lee at al., 2018). However, the existence of resources, such as family support, positively impacts quality of life, showing that there is a positive correlation between quality of life and perceived social support in caregivers of cancer patients (Lee et al., 2017; Pedraza & González, 2015). Also, a previous report confirms that caregivers who perceive low social support from their family show less well‐being compared to those who perceive high social support (Muñoz et al., 2015). In this line of research, social support is a good method to preserve the quality of life in cancer caregivers, since it has a mediating effect on health (Burnette et al., 2017). A cross‐sectional study by Burnette et al., (2017) with informal caregivers in Albania found that social support mediates the negative relationship between distress and quality of life. Caregivers of children with leukaemia in Turkey who perceived a high level of social support were better able to meet their basic needs and showed low levels of psychological symptoms (Demirtepe‐Saygılı & Bozo, 2011).

Despite these results, studies about the mediating role of perceived emotional support between anxiety and depression and quality of life of caregivers are limited. Previous research suggests that quality of life might be deteriorated due to exhaustion of caregiving tasks during the first months after cancer diagnosis (El‐Jawahri et al., 2015; Lee et al., 2018). In addition, lack of emotional expression and emotional inhibition might be related to inadequate coping skills and negative impact when facing a stressful situation such as cancer diagnosis in a close relative. Conversely, the expression of emotions may help to reach a better adaptation to the situation, so the existence of a good social network that understands the needs and provides emotional support may facilitate a successful adaptation to such events (Zachariae, 2020). This is particularly relevant in cancer caregivers in this period of time, when the impact of caregiving is different depending on the illness phase (Marziliano & Moyer, 2020).

Therefore, this study examines the mediation capacity that perceived emotional support can have in T1 (45–60 days after diagnosis) and T2 (180–200 days after) between depression and anxiety in T1 and quality‐of‐life subscales in T2. The hypothesis was that high levels of anxiety and depression in T1 would be related to worse quality of life in T2, and this relationship would be mediated by the emotional support perceived at the beginning of the diagnosis and six months later.

## 2 Methods

### 2.1 Participants

The aim of this research was to establish the mediator role of perceived emotional support between anxiety, depression and quality of life in two different points T1 (45–60 days after diagnosis) and T2 (180–200 days after) in a sample of caregivers of cancer patients. The participants were 67 informal caregivers of cancer patients with a confirmed diagnosis of cancer participated in this study. Inclusion criteria were aged between 21–65 years old; with no previous history of cancer or mental health illness; no previous medical history of dementia or intellectual disability; currently in a relation (partner/father/mother/son/daughter/friend/brother/sister) with a patient in the last 30–45 days who were qualified for treatment (surgical, chemotherapy or radiotherapy); able to read and understand the questionnaires to complete it adequately; living in the same home as the patient and no professional training in the care of chronic diseases. This study is longitudinal, ex post facto prospective (Montero & León, 2002), with convenience sampling.

### 2.2 Instruments

Participants completed the Medical Outcomes Study 36‐Item Short Form (SF‐36). This instrument assesses the quality of life related to physical and mental health. It is composed of 36 items, divided into eight health dimensions: physical function, physical role, body pain, perception of general health, vitality, social functioning, emotional role and mental health. To obtain the scores, the items that compose each dimension are coded, added and transformed on a scale from 0–100, with higher scores indicating better health outcomes. Psychometric properties have been shown to be valid, reliable and sensitive, with Cronbach's alpha on all scales >0.70 (Vilagut et al., 2005).

The participants also completed the Hospital Anxiety and Depression Scale (HADS). It is an instrument used to detect emotional distress, anxiety and depression in patients with physical illness. It consists of 14 items that are divided into two subscales: HAD‐A for anxiety and HAD‐D for depression. Each item scores from 0–3. The cut‐off points for the interpretation of anxiety and depression are as follows: from 0–7 is normal; 8–10 is probable; and 11–21 is high. Cronbach's alpha analysis shows a high internal consistency in both subscales, with an index >0.80. The test–retest reliability presented a correlation coefficient higher than 0.85 (Quintana et al., 2003; Terol‐Cantero et al., 2015). This instrument has been validated, and it is applied frequently in research with cancer caregivers (González et al., 2020; Gough & Hudson, 2009; Vázquez et al., 2015; Wang et al., 2020; Yang et al., 2020). Participants also completed the Berlin Social Support Scale (BSSS). This tool evaluates social support through six scales based on a multidimensional view: perceived available support, need for support, support seeking, actual received support, provided support and protective buffering. It has the possibility to generate three subscales (instrumental, emotional and information support). Perceived emotional support subscale was obtained dividing the perceived available support scale into two subscales: instrumental and emotional support. Perceived emotional support subscale includes items such as “There are some people who truly like me” or “Whenever I am not feeling well, other people show me that they are fond of me.” Scale scores are obtained by adding the sum scores or generating an average scale score. The internal consistency is good, with values between 0.75–0.96 (DiMillo et al., 2019; Schulz & Schwarzer, 2003). Participants also completed a sociodemographic questionnaire including information about gender, age, education, location, profession, type of relationship with the patient, general health, and partner's cancer type and treatment.

### 2.3 Procedure

The recruitment and data collection were performed at the Reina Sofia University Hospital in Córdoba (Spain) between March 2017–November 2018. The researchers contacted with the nursing team of the oncology unit in order to provide them with the inclusion criteria of the participants of the study. Participants were consecutively recruited and prior to the application of the questionnaires, the participants and the patient in their care diagnosed with cancer signed an informed consent form in which the objectives of the study were made explicit and the confidentiality of the results obtained was guaranteed. The first data collection was performed between the first and second month (45–60 days) after the patient obtained the diagnosis of cancer, and the second was six months later (180–200 days). In the study, 176 people were invited to participate, 141 completed the first evaluation (80% response rate), and 67 completed the entire evaluation process (47% response rate); this response rate can be seen in other studies with caregivers (Alfheim et al., 2018; Saria et al., 2017). Reasons for not completing the second evaluation were the end of treatment (*N* = 41), referred to other centres (*N* = 12), palliative care (*N* = 8), not attending the appointment (*N* = 8) and deceased (*N* = 3). The research design was approved by the Portal of Biomedical Research of Andalusia (ref. 3.262).

### 2.4 Statistical analysis

All analyses were performed with the IBM SPSS Statistics Version 22.0 for Windows statistical package. The confidence interval (CI) used was 95%. First, descriptive statistics were obtained for the sociodemographic variables of informal caregivers, as well as for the variables of perceived emotional support, anxiety, depression and quality of life. Then, a Spearman correlation analysis was carried out to test the relationships between the variables. Finally, the PROCESS models 4 was used to examine the mediation effects of perceived emotional support in T1/T2 in the relationship between depression T1 and quality‐of‐life T2. There were no missing data. The estimates of the indirect effects were based on running 10,000 bootstrap iterations of computed samples at 95% CI. Results are considered significant with *p* < .05.

## 3 RESULTS

Sociodemographic data are shown in Table 1. The normality assumption was verified to perform the correlation analysis through the Kolmogorov–Smirnov test in the variables of anxiety, depression and social support in T1 and in the variables related to quality of life and social support in T2. None of the variables fulfilled this assumption (*p* < .05); therefore, the Spearman correlation coefficient was used to determine the relationship between these variables. The results obtained showed that anxiety in T1 is related to the quality‐of‐life subscales of vitality, emotional role and mental health in T2 (Table 2). It shows no relation to perceived emotional support. Depression in T2 is related to most quality‐of‐life subscales in T2 (physical role, general health, vitality, social function, emotional role and mental health) and also with perceived emotional support in T1 and T2. On the other hand, the emotional support perceived in T1 is related to subscales of quality of life in T2 (body pain, general health, social function, emotional role and mental health). Emotional support perceived in T2 is related to all quality‐of‐life subscales in T2.

**TABLE 1:** Sociodemographic characteristics of informal caregivers

Variables	*N* (*%*)
Gender	
Male	23 (34.3)
Female	44 (65.7)
Education	
Primary	24 (35.8)
Vocational	18 (26.9)
Secondary	10 (14.9)
University	15 (22.4)
Location	
Village	32 (47.8)
Small town	5 (7.5)
Medium town	5 (7.5)
Big city	25 (37.3)
Employment situation	
Full‐time job	22 (32.8)
Own business	11 (16.4)
Seasonal work	14 (20.9)
Unemployment	13 (19.4)
Rent	1 (1.5)
Retirement	6 (9.0)
Relation with patient	
Partner	39 (58.2)
Father/Mother	2 (3.0)
Son/Daughter	19 (28.4)
Friend	2 (3.0)
Brother/Sister	5 (7.5)
General health	
Very good	7 (10.4)
Good	47 (70.1)
Normal	13 (19.4)
Patient cancer type	
Head and Neck	7 (10.4)
Lung	4 (6.0)
Breast	14 (20.9)
Gastrointestinal	32 (47.8)
Uterine/Ovarian	2 (3.0)
Genitourinary	4 (6.0)
Connective tissue/Skin	2 (3.0)
Patient treatment	
Surgery	8 (11.9)
Chemotherapy	12 (17.9)
Radiotherapy	1 (1.5)
Hormonal	1 (1.5)
Surgery and chemotherapy	28 (41.8)
Surgery and radiotherapy	1 (1.5)
Surgery, chemotherapy and radiotherapy	15 (22.4)

Variables	*M* (*SD*)
Age	58.25 (1.60)

Abbreviations: *M*, Mean; *SD*, standard deviation.

**TABLE 2:** Descriptive statistics and Spearman correlation coefficient of the study variables

Variables	Mean (*SD*)	1	2	3	4	5	6	7	8	9	10	11
1. Anxiety T1	8.24 (4.07)	–	–	–	–	–	–	–	–	–	–	–
2. Depression T1	6.40 (3.92)	0.675**	–	–	–	–	–	–	–	–	–	–
3. Perceived emotional Support T1	32.04 (21.75)	−0.205	−0.406**	–	–	–	–	–	–	–	–	–
4. Physical functioning T2	85.37 (16.61)	−0.167	−0.237	0.196	–	–	–	–	–	–	–	–
5. Physical role T2	82.46 (35.09)	−0.109	−0.259*	0.199	0.389**	–	–	–	–	–	–	–
6. Body pain T2	80.28 (17.79)	−0.068	−0.052	−0.335**	0.467**	0.391**	–	–	–	–	–	–
7. General health T2	58.84 (13.56)	−0.152	−0.354**	0.274*	0.366**	0.327**	0.110	–	–	–	–	–
8. Vitality T2	57.84 (12.59)	−0.288*	−0.414**	0.209	0.345**	0.542**	0.205	0.515**	–	–	–	–
9. Social functioning T2	66.42 (22.21)	−0.228	−0.439**	0.657**	0.205	0.260*	−0.189	0.251*	0.350**	–	–	–
10. Emotional role T2	67.16 (42.44)	−0.258*	−0.364**	0.332**	0.103	0.420**	−0.123	0.403**	0.444**	0.408**	–	–
11. Mental health T2	58.69 (14.14)	−0.364**	−0.453**	0.348**	0.156	0.333**	0.082	0.497**	0.669**	0.521**	0.336**	–
12. Perceived emotional Support T2	31.75 (21.49)	−0.105	−0.278*	0.877**	0.130	0.301*	−0.40**	0.334**	0.274*	0.641**	0.432**	0.382**

Abbreviation: *SD*, standard deviation.

* *p* <.5; **p < .01.

The results of mediation analysis (Tables 3 and 4) show that depression in T1 indirectly influenced social function, emotional role and emotional health in T2 through perceived emotional support in T1, but did not affect general health in T2. On the other hand, the results show that depression in T1 indirectly influenced physical role, social function, emotional role and mental health in T2 through the perceived emotional support in T2, but not in general health or vitality in T2.

**TABLE 3:** Mediation analysis for depression and quality of life with perceived emotional support T1 as mediator

Model	*β*	*SE*	*t*	*p*	CI
Direct effects					
Depression T1 → Perceived emotional support T1	−1.79	0.652	−2.75	.007*	[−3.09, −0.489]^a^
Depression T1 → General Health T2	−1.19	0.415	−2.87	.005*	[−2.02, −0.363]^a^
Perceived emotional support T1 → General Health T2	0.096	0.075	1.28	.204	[−0.053, 0.245]
Depression T1 → Social Functioning T2	−1.35	0.509	−2.65	.010*	[−2.37, −0.335]^a^
Perceived emotional support T1 → Social Functioning T2	0.634	0.092	6.91	.000*	[.451, 0.817]^a^
Depression T1 → Emotional Role T2	−2.70	1.29	−2.08	.041*	[−5.29, −0.109]^a^
Perceived emotional support T1 → Emotional Role T2	0.519	0.234	2.22	.029*	[.053, 0.987]^a^
Depression T1 → Mental Health T2	−1.25	0.405	−3.07	.003*	[−2.05, −0.437]^a^
Perceived emotional support T1 → Mental Health T2	0.194	0.073	2.66	.009*	[.048, 0.340]^a^
Indirect effects					
Depression T1 → Perceived emotional support T1 → General Health T2	−0.172	−	−	−	[−0.643, 0.097]
Depression T1 → Perceived emotional support T1 → Social Functioning T2	−1.14	−	−	−	[−2.08, −0.320]^a^
Depression T1 → Perceived emotional support T1 → Emotional Role T2	−0.932	−	−	−	[−2.50, −0.058]^a^
Depression T1 → Perceived emotional support T1 → Mental Health T2	−0.349	−	−	−	[−0.831, −0.051]^a^

Abbreviations: CI, confidence interval; SE, standard error.

^a^ Indicate that bootstrapped confidence interval does not go through zero.

* *p* <.5.

**TABLE 4:** Mediation analysis for depression and quality of life with perceived emotional support T2 as mediator

Model	*β*	*SE*	*t*	*p*	CI
Direct effects					
Depression T1 → Perceived emotional support T2	−1.63	0.650	−2.51	.014^a^, *	[−2.93, −0.337]^a^, ^*^
Depression T1 → Physical Role T2	−0.186	1.12	−0.164	.869	[−2.44, 2.07]
Perceived emotional support T2 → Physical Role T2	0.431	0.205	2.09	.040^a^, *	[.019, 0.842]^a^, ^*^
Depression T1 → General Health T2	−1.19	0.410	−2.89	.005^a^, *	[−2.01, −0.368]^a^, ^*^
Perceived emotional support T2→ General Health T2	0.107	0.074	1.44	.153	[−0.041, 0.257]
Depression T1 → Vitality T2	−1.10	0.385	−2.86	.005^a^, *	[−1.87, −0.334]^a^, ^*^
Perceived emotional support T2 → Vitality T2	0.078	0.070	1.11	.271	[−0.062, 0.218]
Depression T1→ Social Functioning T2	−1.45	0.506	−2.86	.005^a^, *	[−2.46, −0.440]^a^, ^*^
Perceived emotional support T2 → Social Functioning T2	0.633	0.092	6.86	.000^a^, *	[.449, 0.817]^a^, ^*^
Depression T1 → Emotional Role T2	−2.66	1.27	−2.09	.040^a^, *	[−5.20, −0.117]^a^, ^*^
Perceived emotional support T2 → Emotional Role T2	0.596	0.231	2.57	.012^a^, *	[.133, 1.05]^a^, ^*^
Depression T1 → Mental Health T2	−1.26	0.401	−3.16	.002^a^, *	[−2.07, −0.466]^a^, ^*^
Perceived emotional support T2 → Mental Health T2	0.202	0.073	2.766	.007^a^, *	[.056, 0.347]^a^, ^*^
Indirect effects					
Depression T1 → Perceived emotional support T2 →Physical Role T2	−0.704	−	−	−	[−1.71, −0.031]^a^, ^*^
Depression T1 → Perceived emotional support T2 → General Health T2	−0.176	−	−	−	[−0.590, 0.069]
Depression T1 → Perceived emotional support T2 → Vitality T2	−0.127	−	−	−	[−0.449, 0.071]
Depression T1 → Perceived emotional support T2 → Social Functioning T2	−1.03	−	−	−	[−1.89, −0.232]^a^, ^*^
Depression T1 → Perceived emotional support T2 → Emotional Role T2	−0.976	−	−	−	[−2.38, −0.097]^a^, ^*^
Depression T1 → Perceived emotional support T2 → Mental Health T2	−1.26	−	−	−	[−2.06, −0.466]^a^, ^*^

Abbreviations: CI, confidence interval; *SE*, standard error.

^a^ Indicate that bootstrapped confidence interval does not go through zero.

* *p* < .5.

## 4 DISCUSSION

Caregivers of cancer patients often have a deterioration of quality of life and social support along with anxiety and depressive symptoms (Bauer et al., 2018; Geng et al., 2018). Previous studies showed that psychological distress in terms of anxiety, depression and social support are variables that are related to quality of life (Burnette et al., 2017; Chandlyden et al., 2016; Delalibera et al., 2015). In this study, the results obtained from the bivariate correlations showed that anxiety and depression, at the beginning of the diagnosis, are negatively related to quality of life six months later in the vitality, emotional role and mental health subscales. In addition, depression was also related to physical role, general health and social function. These results are in line with previous research that shows that anxiety and depression negatively influence the quality of life of caregivers in this period of time, particularly in the domains highlighted (Qiuping et al., 2018). Depression at the beginning of the diagnosis is also negatively related to perceived emotional support after diagnosis and six months later. The relationship between depression and social support is well documented, showing that caregivers that obtained support from family members, friends or medical staff reported a low level of depression, as other people may help to reframing the cancer experience through different perceptions and attitudes towards a stressful experience (Balfe et al., 2016; Santini et al., 2015; Xyaoyun & Fenglan, 2020).

On the other hand, emotional support at the beginning of the diagnosis is related to body pain, general health, social functioning, emotional role and mental health, and at six months, it is also related to physical role and vitality. This suggests that as care is prolonged, perceived emotional support is more important because generally the need to release emotions and fears to other people increases due to all the changes made to adapt to disease (García et al., 2016). In this line, regarding the mediation analysis, the results show that the emotional support perceived at the beginning of the diagnosis mediates the relationship between depression and social function, emotional role and mental health; and perceived emotional support six months after diagnosis also mediates the relationship between depression and the same domains as T1 (social function, emotional role and mental health) and physical role. The physical role subscale collected information about the role limitations due to physical health problems, including impairments in daily life activities and work. These results are in line with previous research and are probably related to the fact that social support promotes adaptive health behaviours and provides a feeling of well‐being that reduces the negative effects of the stressful situation in different aspects (García et al., 2016; Nightingale et al., 2016; Santini et al., 2015). It should be taken into account that from the moment of diagnosis and throughout the disease process, caregivers find themselves in a situation of uncertainty that generates a certain emotional distress and lower quality of life and this situation may worsen over time (Nightingale et al., 2016). According to the stress buffer hypothesis, a high level of discomfort will generate a greater importance of social support to reduce the negative effects on health‐related outcomes (Zachariae, 2020). This hypothesis may explain the mediation effect of perceived emotional support between depression and diverse facets of quality of life of informal caregivers at two different measurement points. Moreover, the fact that this mediation effect on physical role appeared at T2 but not at T1 reinforces the idea that the importance of emotional support may increase over time and display its buffering effects on other facets of quality of life as the disease progresses. These data suggest that it is necessary to pay attention to the psychological evaluation in terms of anxiety, depression and social support not only of patients but also of caregivers at the beginning of the disease, with the objective of preventing changes in the quality of life that can appear in the first six months after a cancer diagnosis and developing interventions to prevent reduced quality of life during this period.

Finally, it is necessary to mention some limitations that may influence the results obtained in the present study. First, the sample size is relatively limited, although it is similar to recent studies with caregivers (Kim et al., 2016; Terro & Crean, 2017). On the other hand, the inclusion of patients with different types of cancer is an aspect to take into account because different types of cancer can give rise to different needs of the patient that have to be covered by the help provided by the caregiver. In addition, a significant loss of participants was observed due to different causes, which is reasonable considering the type of patients who attended a provincial hospital, who were later referred on most occasions to other centres closer to their residence.

The results obtained may be useful to demonstrate the importance of evaluating and detecting emotional distress in informal caregivers in the early stages of the disease of cancer patients and thus to be able to design preventive strategies promoting social support that can improve the quality of life in this population during the course of the disease.

## CONFLICT OF INTEREST

The authors declare no conflict of interest.

## AUTHOR CONTRIBUTIONS

MGC, FGT, MJ, AGS, MJJ, JAM, MJM and EA: Conceptualization and study design. FGT, AGS, MJJ and MJM: Data collection. MGC, FGT, JAM: Data Analysis. MGC, FGT, MJ, MJJ, JAM, EA: Data interpretation. MGC, FGT, MJ, JAM, EA: Writing draft and revision. All authors revised the final manuscript. Margarita García‐Carmona and Francisco García‐Torres, should be considered joint first author.

## ETHICAL APPROVAL

The research was approved by the Portal of Biomedical Research of Andalusia (ref. 3.262).

## Data Availability

The data that support the findings of this study are available from the corresponding author upon reasonable request.

## References

[nop2887-bib-0001] Alfheim, H. B. , Småstuen, M. C. , Hofsø, K. , Tøien, K. , Rosseland, L. A. , & Rustøen, T. (2018). Quality of life in family caregivers of patients in the intensive care unit: A longitudinal study. Australian Critical Care, 32(6), 479–485. 10.1016/j.aucc.2018.09.005 30503245

[nop2887-bib-0002] American Cancer Society (2016). What is a cancer caregiver?. https://www.cancer.org/treatment/caregivers/what‐a‐caregiver‐does/who‐and‐what‐are‐caregivers.html

[nop2887-bib-0003] Asociación Española Contra el Cáncer (2019). Informe sobre la atención psicológica a pacientes de cáncer y familiares en España. Observatorio del Cáncer AECC. http://www.infocoponline.es/pdf/Atencion_psicologica.pdf

[nop2887-bib-0004] Balfe, M. , O'Brien, K. , Timmons, A. , Butow, P. , O'Sullivan, E. , Gooberman‐Hill, R. , & Sharp, L. (2016). What factors are associated with posttraumatic growth in head and neck cancer carers? European Journal of Oncology Nursing, 21, 31–37. 10.1016/j.ejon.2015.11.005 26952676

[nop2887-bib-0005] Bauer, M. R. , Bright, E. E. , MacDonald, J. J. , Cleary, E. H. , Hines, O. J. , & Stanton, A. L. (2018). Quality of life in patients with pancreatic cancer and their caregivers: A systematic review. Pancreas, 47(4), 368–375. 10.1097/mpa.0000000000001025 29521939

[nop2887-bib-0006] Bray, F. , Ferlay, J. , Soerjomataram, I. , Siegel, R. L. , Torre, L. A. , & Jemal, A. (2018). Global cancer statistics 2018: GLOBOCAN estimates of incidence and mortality worldwide for 36 cancers in 185 countries. CA: A Cancer Journal for Clinicians, 68(6), 394–424. 10.3322/caac.21492 30207593

[nop2887-bib-0007] Burnette, D. , Duci, V. , & Dhembo, E. (2017). Psychological distress, social support, and quality of life among cancer caregivers in Albania. Psycho‐Oncology, 26(6), 779–786. 10.1002/pon.4081 26810582

[nop2887-bib-0008] Chandylen, L. N. , Curbow, B. A. , Wingard, J. R. , Pereira, D. B. , & Carnaby, G. (2016). Burden, quality of life, and social support in caregivers of patients undergoing radiotherapy for head and neck cancer: A pilot study. Chronic Illness, 12(3), 236–243. 10.1177/1742395316644305 27068111 PMC5515480

[nop2887-bib-0009] De Padova, S. , Casadei, C. , Berardi, A. , Bertelli, T. , Filograna, A. , Cursano, M. C. , Menna, C. , Burgio, S. L. , Altavilla, A. , Schepisi, G. , Prati, S. , Montalti, S. , Chovanec, M. , Banna, G. L. , Grassi, L. , Mego, M. , & De Giorgi, U. (2019). Caregiver emotional burden in testicular cancer patients: From patient to caregiver support. Frontiers in Endocrinology, 10(318), 1–7. 10.3389/fendo.2019.00318 31191451 PMC6546807

[nop2887-bib-0010] Delalibera, M. , Presa, J. , Barbosa, A. , & Leal, I. (2015). Sobrecarga no cuidar e suas repercussões nos cuidadores de pacientes em fim de vida: revisão sistemática da literatura. Ciência & Saúde Coletiva, 20(9), 2731–2747. 10.1590/1413-81232015209.09562014 26331505

[nop2887-bib-0011] Demirtepe‐Saygılı, D. , & Bozo, Ö. (2011). Perceived social support as a moderator of the relationship between caregiver well‐being indicators and psychological symptoms. Journal of Health Psychology, 16(7), 1091–1100. 10.1177/1359105311399486 21459922

[nop2887-bib-0012] DiMillo, J. , Hall, N. C. , Ezer, H. , Schwarzer, R. , & Körner, A. (2019). The Berlin Social Support Scales: Validation of the Received Support Scale in a Canadian sample of patients affected by melanoma. Journal of Health Psychology, 1, 1785–1795. 10.1177/1359105317700968 28810441

[nop2887-bib-0013] El‐Jawahri, A. R. , Traeger, L. N. , Kuzmuk, K. , Eusebio, J. R. , Vandusen, H. B. , Shin, J. A. , Keenan, T. , Gallagher, E. R. , Greer, J. A. , Pirl, W. F. , Jackson, V. A. , Ballen, K. K. , Spitzer, T. R. , Graubert, T. A. , McAfee, S. L. , Dey, B. R. , Chen, Y. B. , & Temel, J. S. (2015). Quality of life and mood of patients and family caregivers during hospitalization for hematopoietic stem cell transplantation. Cancer, 121(6), 951–959. 10.1002/cncr.29149 25469752 PMC4352120

[nop2887-bib-0014] García, F. E. , Manquián, E. , & Rivas, G. (2016). Bienestar psicológico, estrategias de afrontamiento y apoyo social en cuidadores informales. Psicoperspectivas, 15(3), 87–97. 10.5027/PSICOPERSPECTIVAS-VOL15-ISSUE3-FULLTEXT-789

[nop2887-bib-0015] Geng, H. M. , Chuang, D. M. , Yang, F. , Yang, Y. , Liu, W. M. , Liu, L. H. , & Tian, H. M. (2018). Prevalence and determinants of depression in caregivers of cancer patients: A systematic review and meta‐analysis. Medicine, 97(39), e11863. 10.1097/MD.0000000000011863 30278483 PMC6181540

[nop2887-bib-0016] González, C. P. , Roman‐Calderon, J. P. , & Limonero, J. T. (2020). The relationship between positive aspects of caring, anxiety and depression in the caregivers of cancer patients: The mediational role of burden. European Journal of Cancer Care, 30, e13346. 10.1111/ecc.13346 33037849

[nop2887-bib-0017] Gough, K. , & Hudson, P. (2009). Psychometric properties of the Hospital Anxiety and Depression Scale in family caregivers of palliative care patients. Journal of Pain and Symptom Management, 37(5), 797–806. 10.1016/j.jpainsymman.2008.04.012 18789643

[nop2887-bib-0018] Kim, J. Y. , Sun, V. , Raz, D. J. , Williams, A. C. , Fujinami, R. , Reckamp, K. , Koczywas, M. , Cristea, M. , Hurria, A. , & Ferrell, B. (2016). The impact of lung cancer surgery on quality of life trajectories in patients and family caregivers. Lung Cancer, 101, 35–39. 10.1016/j.lungcan.2016.08.011 27794406 PMC5123701

[nop2887-bib-0019] Lambert, S. , Girgis, A. , Descallar, J. , Levesque, J. V. , & Jones, B. (2017). Trajectories of mental and physical functioning among spouse caregivers of cancer survivors over the first five years following the diagnosis. Patient Education and Counseling, 100(6), 1213–1221. 10.1016/j.pec.2016.12.031 28089132

[nop2887-bib-0020] Lee, C.‐Y. , Lee, Y. U. , Wang, L.‐J. , Chien, C.‐Y. , Fang, F.‐M. , & Lin, P.‐Y. (2017). Depression, anxiety, quality of life, and predictors of depressive disorders in caregivers of patients with head and neck cancer: A six‐month follow‐up study. Journal of Psychosomatic Research, 100, 29–34. 10.1016/j.jpsychores.2017.07.002 28789790

[nop2887-bib-0021] Lee, Y. , Lin, P. Y. , Chien, C. Y. , Fang, F. M. , & Wang, L. J. (2018). A comparison of psychological well‐being and quality of life between spouse and non‐spouse caregivers in patients with head and neck cancer: A 6‐month follow‐up study. Neuropsychiatric Disease and Treatment, 14, 1697–1704. 10.2147/NDT.S162116 29988736 PMC6029606

[nop2887-bib-0022] Li, Q. , Xu, Y. , Zhou, H. , & Loke, A. Y. (2016). Factors influencing the health‐related quality of life of Chinese advanced cancer patients and their spousal caregivers: A cross‐sectional study. BMC Palliative Care, 15(1), 1–14. 10.1186/s12904-016-0142-3 27484209 PMC4971682

[nop2887-bib-0023] Marziliano, A. , & Moyer, A. (2020). Caregivers and clinical health psychology. The Wiley Encyclopedia of Health Psychology, 9–17.

[nop2887-bib-0024] McDonald, J. , Swami, N. , Pope, A. , Hales, S. , Nissim, R. , Rodin, G. , Hannon, B. , & Zimmermann, C. (2018). Caregiver quality of life in advanced cancer: Qualitative results from a trial of early palliative care. Palliative Medicine, 32(1), 69–78. 10.1177/0269216317739806 29130418

[nop2887-bib-0025] Montero, I. , & León, O. G. (2002). Clasificación y descripción de las metodologías de investigación en Psicología. International Journal of Clinical and Health Psychology, 2(3), 503–508.

[nop2887-bib-0026] Moreno‐González, M. M. , Galarza‐Tejada, D. M. , & Tejada‐Tayabas, L. M. (2019). Experiencias del cuidado familiar durante el cáncer de mama: La perspectiva de los cuidadores. Revista Da Escola De Enfermagem Da USP, 53, artículo e03466. 10.1590/s1980-220x2018012203466 31482950

[nop2887-bib-0027] Muñoz, C. P. , Nieto, B. B. , Méndez, M. J. M. , Morillejo, E. A. , & Carrique, N. C. (2015). Repercusiones psicosociales del cáncer infantil: Apoyo social y salud en familias afectadas. Revista Latinoamericana De Psicología, 47(2), 93–101. 10.1016/j.rlp.2014.07.002

[nop2887-bib-0028] Nightingale, C. L. , Curbow, B. A. , Wingard, J. R. , Pereira, D. B. , & Carnaby, G. D. (2016). Burden, quality of life, and social support in caregivers of patients undergoing radiotherapy for head and neck cancer: A pilot study. Chronic Illness, 12(3), 236–245. 10.1177/1742395316644305 27068111 PMC5515480

[nop2887-bib-0029] Pedraza, H. M. P. , & González, G. M. C. (2015). Calidad de vida y soporte social en los cuidadores familiares de personas en tratamiento contra el cáncer. Revista De La Universidad Industrial De Santander. Salud, 47(2), 125–136.

[nop2887-bib-0030] Peh, C. X. , Liu, J. , & Mahendran, R. (2020). Quality of life and emotional distress among caregivers of patients newly diagnosed with cancer: Understanding trajectories across the first year post‐diagnosis. Journal of Psychosocial Oncology, 1–16, 10.1080/07347332.2020.1760994 32367769

[nop2887-bib-0031] Qiuping, L. , Yi, L. , Yinghua, X. , & Huiya, Z. (2018). The impact of depression and anxiety on quality of life in Chinese cancer patient‐family caregiver dyads, a cross‐sectional study. Health and Quality of Life Outcomes, 16(230), 1–15. 10.1186/s12955-018-1051-3 30545383 PMC6293618

[nop2887-bib-0032] Quintana, J. M. , Padierna, A. , Esteban, C. , Arostegui, I. , Bilbao, A. , & Ruiz, I. (2003). Evaluation of the psychometric characteristics of the Spanish version of the Hospital Anxiety and Depression Scale. Acta Psychiatrica Scandinavica, 107(3), 216–221. 10.1034/j.1600-0447.2003.00062.x 12580829

[nop2887-bib-0033] Sánchez, R. , Sierra, F. A. , & Martín, E. (2015). ¿Qué es calidad de vida para un paciente con cáncer? Avances En Psicología Latinoamericana, 33(3), 371–385. 10.12804/apl33.03.2015.01

[nop2887-bib-0034] Santini, Z. I. , Koyanagi, A. , Tyrovolas, S. , Mason, C. , & Haro, J. M. (2015). The association between social relationships and depression: A systematic review. Journal of Affective Disorders, 175, 53–65. 10.1016/j.jad.2014.12.049 25594512

[nop2887-bib-0035] Saria, M. , Courchesne, N. , Evangelista, L. , Carter, J. , MacManus, D. , Gorman, M. K. , Nyamathi, A. , Phillips, L. , Piccioni, D. , Kesari, S. , & Maliski, S. (2017). Anxiety and depression associated with burden in caregivers of patients with brain metastases. Oncology Nursing Forum, 44(3), 306–315. 10.1188/17.ONF.306-315 28635984 PMC10188286

[nop2887-bib-0036] Schulz, U. , & Schwarzer, R. (2003). Soziale unterstüzung bei der Krankheitsbewäsltigung. Die Berliner Social Support Skalen (BSSS) [Social support in coping with illness: The Berlin Social Support Scales (BSSS)]. Diagnostica, 49, 73–82.

[nop2887-bib-0037] Sklenarova, H. , Krümpelmann, A. , Haun, M. W. , Friederich, H.‐C. , Huber, J. , Thomas, M. , Winkler, E. C. , Herzog, W. , & Hartmann, M. (2015). When do we need to care about the caregiver? Supportive care needs, anxiety, and depression among informal caregivers of patients with cancer and cancer survivors. Cancer, 121(9), 1513–1519. 10.1002/cncr.29223 25677095

[nop2887-bib-0038] Sociedad Española de Oncología Médica (2020). Las cifras del cáncer en España 2020. https://seom.org/seomcms/images/stories/recursos/Cifras_del_cancer_2020.pdf

[nop2887-bib-0039] Stanton, A. L. , Rowland, J. H. , & Ganz, P. A. (2015). Life after diagnosis and treatment of cancer in adulthood: Contributions from psychosocial oncology research. American Psychologist, 70(2), 159–174. 10.1037/a0037875 25730722

[nop2887-bib-0040] Tan, J. Y. , Molassiotis, A. , Lloyd‐Williams, M. , & Yorke, J. (2018). Burden, emotional distress and quality of life among informal caregivers of lung cancer patients: An exploratory study. European Journal of Cancer Care, 27(1), artículo e12691. 10.1111/ecc.12691 28417550

[nop2887-bib-0041] Terol‐Cantero, M. C. , Cabrera‐Perona, V. , & Martín‐Aragón, M. (2015). Revisión de estudios de la Escala de Ansiedad y Depresión Hospitalaria (HAD) en muestras españolas. Anales De Psicología, 31(2), 494–503. 10.6018/analesps.31.2.172701

[nop2887-bib-0042] Terro, W. , & Crean, S. J. (2017). Prospective, longitudinal assessment of quality of life in patients with cancer of the head and neck and their primary carers. British Journal of Oral and Maxillofacial Surgery, 55(6), 613–617. 10.1016/j.bjoms.2017.04.004 28456448

[nop2887-bib-0043] Vázquez, Ó. G. , García, A. M. , Gómez, Á. H. , Tinoco, M. D. R. C. , & Ponce, J. L. A. (2015). Escala hospitalaria de ansiedad y depresión (HADS) en cuidadores primarios informales de pacientes con cáncer: Propiedades Psicométricas. Psicooncología: Investigación Y Clínica Biopsicosocial En Oncología, 12(2), 383–392. 10.5209/rev_PSIC.2015.v12.n2-3.51016

[nop2887-bib-0044] Vilagut, G. , Ferrer, M. , Rajmil, L. , Rebollo, P. , Permanyer‐Miralda, G. , Quintana, J. M. , Santed, R. , Valderas, J. M. , Ribera, A. , Domingo‐Salvany, A. , & Alonso, J. (2005). El cuestionario de salud SF‐36 español: Una década de experiencia y nuevos desarrollos. Gaceta Sanitaria, 19(2), 135–150. 10.1157/13074369 15860162

[nop2887-bib-0045] Wang, T. , Molassiotis, A. , Tan, J. Y. , Chung, B. P. M. , & Huang, H. Q. (2020). Prevalence and correlates of unmet palliative care needs in dyads of Chinese patients with advanced cancer and their informal caregivers: A cross‐sectional survey. Supportive Care in Cancer, 29(3), 1683–1698. 10.1007/s00520-020-05657-w 32776164

[nop2887-bib-0046] Xiaoyun, C. , & Fenglan, L. (2020). The relationships among insecure attachment, social support and psychological experiences in family caregivers of cancer inpatients. European Journal of Oncology Nursing, 44, 101691. 10.1016/j.ejon.2019.101691 31751851

[nop2887-bib-0047] Yang, M. , Ma, F. , Lan, B. , Cai, J. , Sun, X. , & Xu, B. (2020). Validity of distress thermometer for screening of anxiety and depression in family caregivers of Chinese breast cancer patients receiving postoperative chemotherapy. Chinese Journal of Cancer Research, 32(4), 476. 10.21147/j.issn.1000-9604.2020.04.05 32963460 PMC7491542

[nop2887-bib-0048] Zachariae, R. (2020). Social relations and health. In J. Sholl & S. I. Rattan (Eds.), Explaining health across the sciences. Healthy ageing and longevity (Vol. 12, pp. 383–403). Springer. 10.1007/978-3-030-52663-4_22

